# Paeonol Attenuates Hepatic Ischemia/Reperfusion Injury by Modulating the Nrf2/HO-1 and TLR4/MYD88/NF-κB Signaling Pathways

**DOI:** 10.3390/antiox11091687

**Published:** 2022-08-29

**Authors:** Mohamed A. Morsy, Yasmine F. Ibrahim, Sara Mohamed Naguib Abdel Hafez, Nagwa M. Zenhom, Anroop B. Nair, Katharigatta N. Venugopala, Pottathil Shinu, Seham A. Abdel-Gaber

**Affiliations:** 1 Department of Pharmaceutical Sciences, College of Clinical Pharmacy, King Faisal University, Al-Ahsa 31982, Saudi Arabia; 2 Al Bilad Bank Scholarly Chair for Food Security in Saudi Arabia, the Deanship of Scientific Research, the Vice Presidency for Graduate Studies and Scientific Research, King Faisal University, Al-Ahsa 31982, Saudi Arabia; 3 Department of Pharmacology, Faculty of Medicine, Minia University, El-Minia 61511, Egypt; 4 Department of Histology and Cell Biology, Faculty of Medicine, Minia University, El-Minia 61511, Egypt; 5 Department of Biochemistry, Faculty of Medicine, Al-Baha University, Albaha 65525, Saudi Arabia; 6 Department of Biochemistry, Faculty of Medicine, Minia University, El-Minia 61511, Egypt; 7 Department of Biomedical Sciences, College of Clinical Pharmacy, King Faisal University, Al-Ahsa 31982, Saudi Arabia

**Keywords:** paeonol, hepatic ischemia/reperfusion, Nrf2/HO-1, TLR4/MYD88/NF-κB/TNF-α, Bax/Bcl-2

## Abstract

Hepatic ischemia/reperfusion (HIR) is the most common type of liver injury following several clinical situations. Modulating oxidative stress and inflammation by Nrf2/HO-1 and TLR4/MYD88/NF-κB pathways, respectively, is involved in alleviating HIR injury. Paeonol is a natural phenolic compound that demonstrates significant antioxidant and anti-inflammatory effects. The present study explored the possible protective effect of paeonol against HIR injury and investigated its possible molecular mechanisms in rats. Rats were randomly divided into four groups: sham-operated control, paeonol-treated sham-operated control, HIR untreated, and HIR paeonol-treated groups. The results confirmed that hepatic injury was significantly aggravated biochemically by elevated serum levels of alanine transaminase and aspartate transaminase, as well as by histopathological alterations, while paeonol reduced the increase in transaminases and alleviated pathological changes induced by HIR. Additionally, paeonol inhibited the HIR-induced oxidative stress in hepatic tissues by decreasing the upraised levels of malondialdehyde and nitric oxide and enhancing the suppressed levels of reduced glutathione and superoxide dismutase activity. Furthermore, paeonol activated the protective antioxidative Nrf2/HO-1 pathway. The protective effect of paeonol was associated with inhibiting the expression of the inflammatory key mediators TLR4, MYD88, NF-κB, and TNF-α. Finally, paeonol inhibited the increased mRNA levels of the pro-apoptotic marker Bax and enhanced the reduced mRNA levels of the anti-apoptotic marker Bcl-2. Taken together, our results proved for the first time that paeonol could protect against HIR injury by inhibiting oxidative stress, inflammation, and apoptosis.

## 1. Introduction

Hepatic ischemia/reperfusion (HIR) injury is the most common type of liver injury following liver resection, hemorrhagic shock, liver transplantation, or trauma, presenting as a sequence of deterioration phenomena and leading to multiple organ failure and even patient death [1,2,3]. It is a complex pathological process that encounters a variety of mechanisms [4]. The restoration of blood supply following liver ischemia is accompanied by intensive production of reactive oxygen species (ROS) and the drooping of antioxidant defenses [5,6]. Consequently, ROS overproduction causes lipid peroxidation, mitochondrial dysfunction, and DNA fragmentation, damaging the cell structure and leading to extensive inflammatory response, necrotic and apoptotic cascade, and ultimately cellular death [7,8].

Using rodent models for HIR induction is essential for better understanding the underlying mechanisms for hepatic injury and possible translational applications [9]. A partial warm HIR model in rats has been widely used in several studies [10,11,12], whereas warm ischemia is clinically associated with hypovolemic shock, hepatic surgery, and liver transplantation and affects mostly the hepatocytes more than the non-parenchymal cells [13]. A cross-clamping is being operated for both the hepatic artery and portal vein for varying lengths of time, leading to a deprivation of approximately 70% of the liver blood flow. Then, restoration of blood supply to the ischemic lobes stimulates superoxide production and changes the liver redox state in favor of a highly oxidative environment. This is followed by the triggering of redox-sensitive transcription factors and proinflammatory cytokines that ultimately results in liver injury [9].

The nuclear factor erythroid-2-related factor 2 (Nrf2) was reported to be crucially correlated with antioxidant defense mechanisms and scavenging of ROS under oxidative stress conditions [14]. Under resting conditions, Nrf2 is bound to Kelch-like ECH-associated protein 1 (Keap-1) and kept in a suppressed state. Upon oxidative stress, Nrf2 dissociates from Keap-1 and translocates to the nucleus, where it binds to the antioxidant response elements (AREs) located in the promoter regions as a battery for detoxifying and antioxidant genes, including heme-oxygenase-1 (HO-1) and superoxide dismutase (SOD) [15,16]. Recent studies have pointed to the therapeutic potential of targeting the Nrf2/HO-1 pathway in HIR injury [17,18].

ROS triggers the stimulation of pivotal signaling molecules, including toll-like receptors (TLRs), which play a key role in regulating inflammatory responses, innate immunity, cell proliferation, and apoptosis [19,20,21]. TLR4, one important member of TLRs, has been reported to play a critical role in the pathogenesis of the brain, kidney, heart, and liver ischemia-reperfusion (IR) injury [10,22,23,24]. The classic signaling cascade of TLR4 includes myeloid differentiation primary response gene 88 (MYD88)-dependent and MYD88-independent pathways, which would cause the stimulation of nuclear factor-kappa B (NF-κB) and triggers the pro-inflammatory cytokines release and apoptosis stimulation [25,26].

Drugs and ischemic pretreatment approaches are commonly utilized in clinical practice in an attempt to prevent HIR injury; however, breakthrough management is still an unmet goal. Several drugs and herbs with antioxidant activities were demonstrated to provide protective effects against HIR injury [27,28,29]. Paeonol (2′-hydroxy-4′-methoxyacetophenone) is a natural phenolic compound that demonstrates significant antioxidant and anti-inflammatory effects on the liver, colon, heart, and stomach [30,31,32,33,34]. The ameliorating effect of paeonol against IR injury was well studied in the heart, brain, and testis [35,36,37]. Therefore, the aim of the current study is to evaluate the protective effect of the pretreatment of paeonol against HIR injury. In order to explore the possible underlying molecular mechanism, we assessed whether the inhibition of key signaling pathway TLR4/MYD88/NF-κB and the reduction of oxidative stress markers and apoptosis contribute to paeonol’s protective effect.

## 2. Materials and Methods

### 2.1. Chemicals and Antibodies

Paeonol was purchased from Sigma-Aldrich Corp. (St. Louis, MO, USA). Alanine transaminase (ALT) and aspartate transaminase (AST) kits were procured from Biodiagnostic (Giza, Egypt). Anti-HO-1 (A19062) was purchased as a rabbit monoclonal antibody from ABclonal (Woburn, MA, USA). Anti-TLR4 (A11226) was purchased as a rabbit polyclonal antibody from ABclonal. Anti-MYD88 (AF5195) was purchased as a rabbit polyclonal antibody from Affinity Biosciences (Jiangsu, China). Anti-NF-κB p65 (AF10036917) was purchased as a rabbit polyclonal antibody from Bioss Antibodies (Woburn, MA, USA). Anti-tumor necrosis factor-α (TNF-α) (AF10036917) was purchased as a rabbit polyclonal antibody from ABclonal. Secondary antibody (ab6734) was purchased as rat polyclonal IgG antibody from Abcam (Cambridge, UK). Other solvents and chemicals were of the highest analytical grade available from their commercial sources.

### 2.2. Animals

The present study was conducted on twenty-eight male Wistar rats. The rats weighed 200–220 g and were purchased from the National Research Center (Giza, Egypt). They were kept in cages and were allowed to get tap water and a commercial rat chow diet for one week before inclusion in the experiment in order to acclimatize to the laboratory environment. The facilities were kept in a 12 h dark:light and maintained at 25 ± 2 °C. The experiment was operated following the ARRIVE ethical guidelines and was approved (317-4-2022) by the Faculty of Medicine-Research Ethics Committee, Minia University, Minya, Egypt.

### 2.3. Experimental Protocol

A total of 28 rats were weighed and divided into four groups (7 rats/group) as follows: Group 1 (sham-operated control group): received the vehicle (0.5% carboxymethyl cellulose (CMC)) *p.o.* once daily for 7 days before the sham operation. Group 2 (paeonol-treated sham-operated group): received paeonol (100 mg/kg) *p.o.* once daily for 7 days before the sham operation. Group 3 (HIR untreated group): received the vehicle (0.5% CMC) *p.o.* once daily for 7 days before the HIR induction. Group 4 (paeonol-treated HIR group): received paeonol (100 mg/kg) *p.o.* once daily for 7 days before the HIR induction. The dose of paeonol was determined according to our preliminary experiments and on the basis of other studies [34].

### 2.4. Induction of HIR

HIR was performed following methods previously used [11,12]. The rats were anesthetized with ketamine (50 mg/kg, *i.m.*), and a midline incision was made in the abdomen in order to isolate the major pedicle, which supplies the median and the left-lateral lobes of the liver that nearly represents 70% of the whole liver volume. Using microvascular bulldog clamp, warm ischemia was rendered for 30 min for the median and the left-lateral lobes. Then, the clamp was removed to permit a reperfusion period of 2 h before euthanasia, while the abdomen was closed by silk sutures. Normal saline was installed at 37 °C into the abdomen every 10 min to avoid tissue dehydration. The rats’ body temperature was maintained at 37 °C using a heating pad. The sham-operated animals, whether paeonol-treated or not, were subjected to the same surgical procedures without vascular occlusion to serve as the negative control group.

### 2.5. Samples Preparation

At the end of the experiment period, blood samples were collected from the neck vessels, then centrifuged for 10 min at 5000 rpm to obtain the sera. The sera were kept at −80 °C for determination of ALT and AST. Furthermore, parts of the liver were homogenized in 20% (weight/volume) ice-cold phosphate buffer (0.01 M, pH 7.4). The liver homogenate was then centrifuged for 20 min at 3000 rpm, and the clear supernatant was aspirated and stored at −80 °C for measurements of biochemical parameters. Other liver samples were fixed in 10% formalin for histopathological and immunohistochemical examinations.

### 2.6. Liver Function Tests

Serum ALT and AST activities were determined using commercially available ALT and AST assay kits following the manufacturer’s instructions.

### 2.7. Oxidative Stress Parameters

Liver oxidative stress markers were determined in the liver homogenate. Malondialdehyde (MDA), the basic lipid peroxidation product, was detected as MDA-thiobarbituric acid pink-colored Schiff base adduct. The colored complex was detected at 535 nm using spectrophotometry and expressed as nmol/g tissue [38]. Nitric oxide (NO) was evaluated colorimetrically by the determination of the accumulation of its stable oxidation end products (nitrite and nitrate). The total nitrite level was detected in the hepatic tissues using the Griess method. The assay is based on the reduction of hepatic nitrate to nitrite through the use of copperized cadmium granules; then, the color develops with Griess reagent in an acidic medium. The final total nitrite level was measured at 540 nm using spectrophotometry, and the results were expressed as nmol/g tissue [39]. Reduced glutathione (GSH) was measured following Ellman’s method, which is based on the production of yellow chromogen, 5-thio-2-nitrobenzoic acid as a result of the reducing effect of the thiol groups of GSH on Ellman’s reagent. This yellow color was measured by the spectrophotometer at 412 nm and expressed as nmol/g tissue [40]. SOD activity in the hepatic homogenates was measured chemically by the method previously prescribed by Marklund and Marklund. One unit of SOD represents the amount of the enzyme that inhibits the pyrogallol autoxidation by 50%. Finally, SOD activity is measured by spectrophotometry at 420 nm [41].

### 2.8. Real-Time Polymerase Chain Reaction (PCR)

The expression of Nrf2, B-cell lymphoma 2 (Bcl-2), and Bcl-2-associated X protein (Bax) genes were conducted by real-time PCR according to the manufacturer’s instructions. Briefly, total RNA was extracted from hepatic tissue by utilizing RiboZol reagent (AMRESCO, Solon, OH, USA) following the manufacturer’s instructions. Then, real-time PCR was conducted with SensiFAST^TM^ SYBR^®^ Hi-ROX One-Step Kit purchased from Meridian Bioscience Inc./Bioline (Memphis, TN, USA) as a reaction of 50 ng RNA template in 25 μL reaction volume having 70 nM of specific primers through the Real-Time PCR Detection System obtained from Kapa Biosystems (Wilmington, MA, USA). The specific sets of the sequence of the primers utilized for Nrf2 [42], Bax [43], Bcl-2 [44], and glyceraldehyde-3-phosphate dehydrogenase (GAPDH) [45] were indicated in Table 1. The SYBR green data analysis was based on a relative quantification of GAPDH, which was used as a reference gene. Calculating the investigated genes’ relative expression level was based on the comparative cycle threshold method [46]. Control samples were set at a value of 1, and each gene’s expression was plotted relative to controls.

### 2.9. Histopathological Examination and Scoring

Liver parts were fixed in formalin (10%) and embedded in paraffin. Serial liver sections were prepared of 5-μm thickness and stained with hematoxylin and eosin [47]. The slides were then blindly examined and photographed using a light microscope (Olympus BX51, Tokyo, Japan) mounting a high-resolution digital camera. The hepatic injury was evaluated based on the degree of inflammatory and apoptotic cells, as well as the degenerated area within the hepatic lobules [48].

### 2.10. Immunohistochemical Examination and Scoring

Liver sections were deparaffinized, rehydrated, and washed with phosphate-buffered saline. First, the endogenous peroxidase activity was blocked using 3% H_2_O_2_. For antigen retrieval, the slides were boiled in 10 mL of citrate buffer (pH 6) using the microwave, then left to be cooled for 20 min at room temperature. Next, the slides were incubated with either HO-1 (1:100), TLR4 (1:100), MYD88 (1:100), NF-κB (1:200), or TNF-α (1:100) for 1 h. Then, detection of the slide sections was conducted using an HRP-conjugated secondary antibody (1:2000), followed by colorimetric detection using a 3,3-diaminobenzidine tetrahydrochloride (DAB) kit. Finally, the sections were rinsed with water before being counterstained with hematoxylin. The mean area fraction of HO-1, TLR4, MYD88, and TNF-α expression was quantified using the ImageJ software in 6 fields for each rat. In addition, the number of NF-κB positive cells was calculated in 6 non-overlapping successive fields under light microscopy, with a high power field equal to 400 [49].

### 2.11. Statistical Analysis

Statistical analysis of the results was conducted using one-way analysis of variance (ANOVA) followed by Tukey’s post-analysis test. Statistical calculations were conducted using GraphPad Prism, version 6.01 for Windows (San Diego, CA, USA). Results were expressed as mean ± SEM. Differences were considered significant with a *p-*value < 0.05.

## 3. Results

### 3.1. Effect of Paeonol on Serum ALT and AST

**Figure 1:**
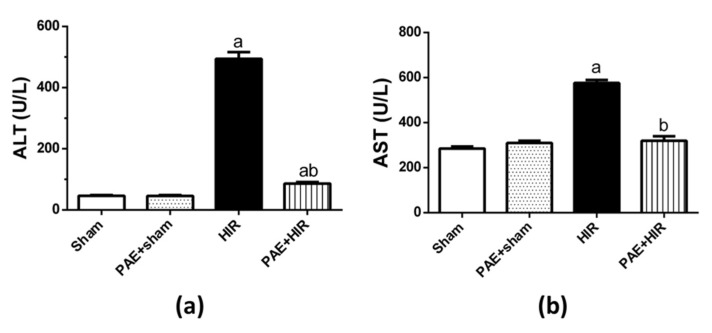
Effect of paeonol (PAE) on liver function tests. Serum activity of (**a**) alanine transaminase (ALT) and (**b**) aspartate transaminase (AST) in hepatic ischemia/reperfusion (HIR) injury in rats. Each value represents the mean ± SEM (*n* = 7). a Significant difference from the sham-operated control group and b significant difference from the HIR group, respectively, at *p* ˂ 0.05.

HIR significantly augmented serum ALT and AST activities compared to the sham-operated control group. Conversely, the pretreatment with paeonol significantly reduced the ALT and AST activities in HIR-induced rats compared to HIR untreated rats. Paeonol treatment does not affect the serum activity of these hepatic enzymes in sham-control rats (Figure 1).

### 3.2. Effect of Paeonol on Hepatic Histopathology

**Figure 2:**
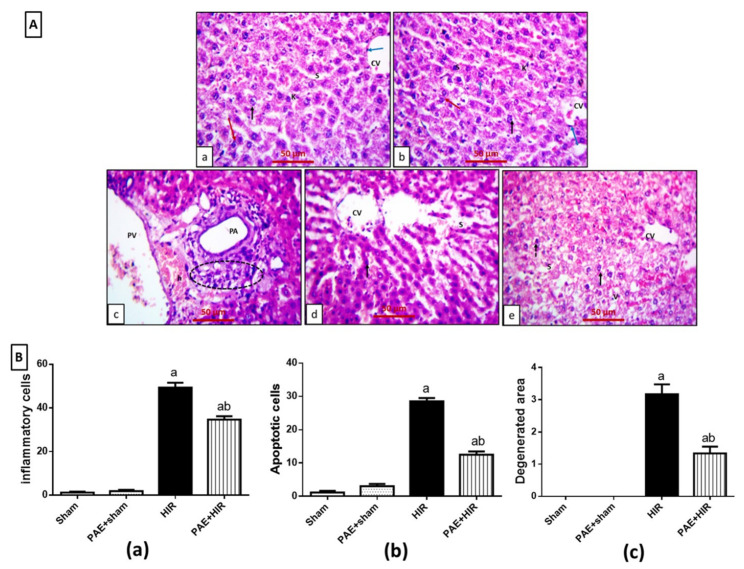
Effect of paeonol (PAE) on histopathological alternations in rats’ hepatic ischemia/reperfusion (HIR) injury. (**A**) Photomicrographs showing sections of rat liver stained with hematoxylin and eosin (×400). (**Aa**,**Ab**) Sham and PAE + sham groups show the same normal lobular architecture with normal central veins (CV). The central veins appeared lined by flat endothelial cells (blue arrows), surrounded by cords of polygonal hepatocytes with granular cytoplasm, and central, rounded, vesicular nuclei (black arrows). Some cells appear binucleated (red arrows). Blood sinusoids (S) are lined by Kupffer cells (K). (**Ac**,**Ad**) The HIR group show disturbed lobular architecture with dilated central veins (CV). Notice the dilated portal artery (PA) and dilated portal vein (PV) with nearby cellular infiltrations (circle) and hemorrhage (h). Cells have darkly stained nuclei with deeply stained cytoplasm (black arrow). Wide blood sinusoids are seen between the hepatocytes (S). (**Ae**) The PAE+HIR group shows normal lobular architecture with apparent normal central veins (CV) except for focal dilated blood sinusoid (S). Hepatocytes appeared polygonal with acidophilic cytoplasm and vesicular nuclei (black arrow) nearly similar to the control. Notice the hepatic cytoplasmic vacuolations (V) and darkly stained nucleus with deeply stained cytoplasm (dashed arrow). (**B**) Semi-quantitative analysis of (**Ba**) inflammatory cells, (**Bb**) apoptotic cells, and (**Bc**) degenerated area within the stained sections. Each value represents the mean ± SEM (*n* = 7). a Significant difference from the sham-operated control group and b significant difference from the HIR group, respectively, at *p* < 0.05.

Rat liver sections of sham and paeonol + sham groups revealed the same normal lobular architecture with normal central veins lined by flatted endothelial cells (Figure 2(Aa,Ab)). These veins were surrounded by numerous cords of polygonal hepatocytes that appeared with granular cytoplasm and central, rounded, vesicular nuclei. Some cells appeared binucleated. The blood sinusoids were lined by Kupffer cells. In contrast, the HIR group displayed disturbed lobular architecture with dilated both central and portal veins. In addition, the sections showed dilated portal arteries and portal veins with nearby cellular infiltrations and hemorrhage. Cells had darkly stained nuclei with deeply stained cytoplasm. Wide blood sinusoids were seen between the hepatocytic cells (Figure 2(Ac,Ad)). Meanwhile, the paeonol + HIR group displayed normal lobular architecture with apparent normal central veins except for focal dilated blood sinusoids. Hepatocytes appeared polygonal with acidophilic cytoplasm and vesicular nuclei nearly similar to the control. In addition, hepatic cytoplasmic vacuolations were seen among the sections (Figure 2(Ae)). The HIR group displayed a significant increase in the number of inflammatory and apoptotic cells, as well as the degenerated areas within the hepatic lobules compared to the sham-operated control group. At the same time, the administration of paeonol significantly mitigated these alternations compared to the HIR group (Figure 2(Ba–Bc)).

### 3.3. Effect of Paeonol on Oxidative Parameters and Antioxidant Profile

#### 3.3.1. Effect of Paeonol on Hepatic MDA, NO, GSH, and SOD

**Figure 3:**
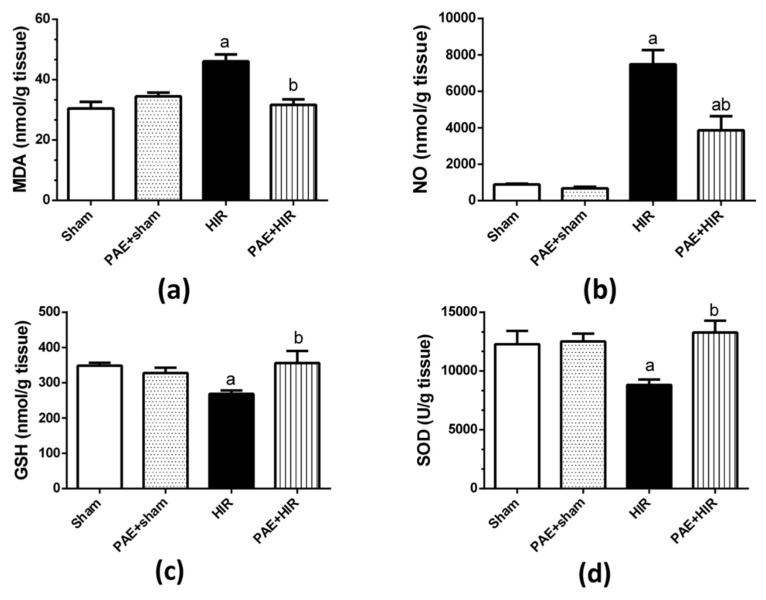
Effect of paeonol (PAE) on hepatic oxidative and antioxidant profile in rats’ hepatic ischemia/reperfusion (HIR) injury. (**a**) Malondialdehyde (MDA) level, (**b**) nitric oxide (NO; total nitrite) level, (**c**) reduced glutathione (GSH) level, and (**d**) superoxide dismutase (SOD) activity. Each value represents the mean ± SEM (*n* = 7). a Significant difference from the sham-operated control group and b significant difference from the HIR group, respectively, at *p* ˂ 0.05.

Next, we evaluated the effect of paeonol on oxidative and antioxidant markers. The data presented in Figure 3 indicated that HIR significantly augmented MDA and NO levels (Figure 3a,b) while significantly reducing GSH level and SOD activity compared to sham-operated control rats (Figure 3c,d). On the other hand, paeonol pretreatment to HIR rats significantly decreased MDA and NO levels and significantly enhanced GSH level and SOD activity compared to HIR untreated rats. Paeonol treatment of the sham-operated rats did not show any effect on any oxidative stress or antioxidant parameter compared to the sham-operated control rats.

#### 3.3.2. Effect of Paeonol on Nrf2/HO-1 Pathway

**Figure 4:**
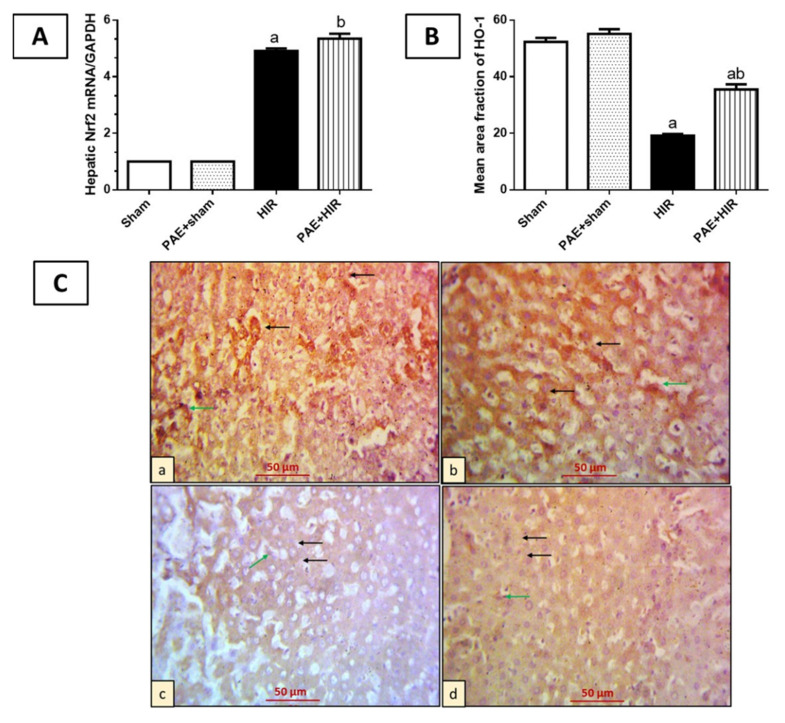
Effect of paeonol (PAE) on the nuclear factor erythroid-2-related factor 2 (Nrf2)/heme-oxygenase-1 (HO-1) pathway in rats’ hepatic ischemia/reperfusion (HIR) injury. (**A**) Relative expression level of the hepatic Nrf2 using real-time polymerase chain reaction. (**B**) Semi-quantification of the hepatic HO-1 expression. (**C**) Photomicrographs of rat liver sections immunohistochemically stained for detection of HO-1 (×400). (**Ca**,**Cb**) Sham and PAE + sham groups show strong, widespread cytoplasmic expression of HO-1 in both hepatocytes (black arrows) and Kupffer cells (green arrows). (**Cc**) The HIR group shows hepatocytes (black arrows) and Kupffer cells (green arrows) with minimal HO-1 cytoplasmic expression. (**Cd**) The PAE + HIR group shows hepatocytes (black arrows) and Kupffer cells (green arrow) with moderate HO-1 cytoplasmic expression. Each value represents the mean ± SEM (*n* = 7). a Significant difference from the sham-operated control group and b significant difference from the HIR group, respectively, at *p* ˂ 0.05.

To investigate the effect of paeonol on crucial antioxidant defense mediators, Nrf2 and HO-1, we further evaluated the mRNA level of Nrf2, and the protein level of HO-1, in rat hepatic tissues. The data presented in Figure 4 demonstrated that HIR significantly increased the mRNA expression of Nrf2 compared to sham-operated control rats, while the pre-administration of paeonol to the HIR rats further significantly enhanced these levels compared to the HIR untreated rats (Figure 4A). On the other hand, immunohistochemical staining of HO-1 revealed that sham and paeonol + sham groups showed hepatocytes and Kupffer cells with cytoplasmic HO-1 expression. Meanwhile, the HIR group exhibited less cytoplasmic expression in hepatocytes and Kupffer cells. However, the paeonol + HIR group exhibited more expression in previously mentioned cells than what was detected in the HIR-induced group (Figure 4B,C).

### 3.4. Effect of Paeonol on Hepatic Inflammatory Mediators

#### 3.4.1. Effect of Paeonol on Hepatic TLR4 and MYD88

**Figure 5:**
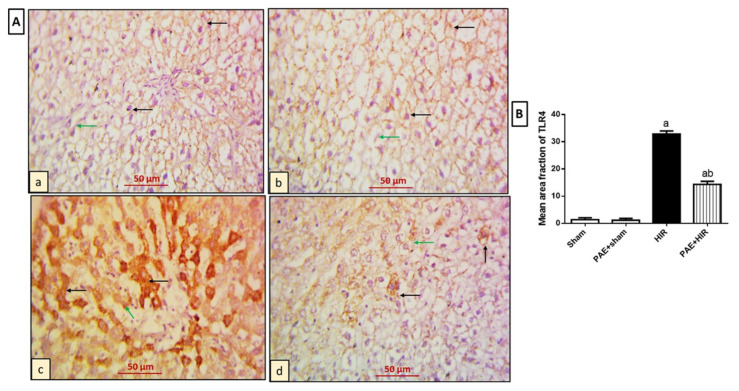
Effect of paeonol (PAE) on toll-like receptor 4 (TLR4) in rats’ hepatic ischemia/reperfusion (HIR) injury. (**A**) Photomicrographs of rat liver sections stained for detection of TLR4 (×400). (**Aa**,**Ab**) Sham and PAE + sham groups show negative expression of TLR4 in both hepatocytes (black arrows) and Kupffer cells (green arrows). (**Ac**) The HIR group shows hepatocytes (black arrows) and Kupffer cells (green arrow) with extensive TLR4 cytoplasmic expression. (**Ad**) The PAE + HIR group shows hepatocytes (black arrows) and Kupffer cells (green arrow) with minimal TLR4 cytoplasmic expression. (**B**) Semi-quantification of the hepatic TLR4 expression. Each value represents the mean ± SEM (*n* = 7). a Significant difference from the sham-operated control group and b significant difference from the HIR group, respectively, at *p* < 0.05.

Previous data demonstrated that TLR4/MYD88 signaling plays a critical role in the occurrence and development of HIR injury [50]. Therefore, we further explored the effects of paeonol on the immunohistochemical staining of TLR4 and MYD88 in hepatic tissues in HIR-induced rats. As regards TLR4 expression (Figure 5A,B), examination of sham and PAE + sham groups showed hepatocytes and Kupffer cells with negative expression (Figure 5(Aa,Ab)). On the other hand, the HIR group showed extensive cytoplasmic expression in the previously mentioned cells compared to the sham-operated control group (Figure 5(Ac)). Meanwhile, the PAE + HIR group displayed less expression in both hepatocytes and Kupffer cells compared to the HIR untreated sections (Figure 5(Ad)).

**Figure 6:**
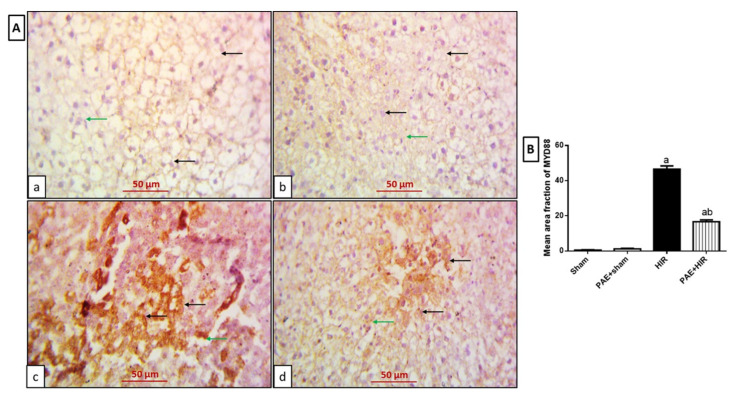
Effect of paeonol (PAE) on myeloid differentiation primary response gene 88 (MYD88) in rats’ hepatic ischemia/reperfusion (HIR). (**A**) Photomicrographs of rat liver sections stained for detection of MYD88 (×400). (**Aa**,**Ab**) Sham and PAE + sham groups show negative expression of MYD88 in both hepatocytes (black arrows) and Kupffer cells (green arrows). (**Ac**) The HIR group shows hepatocytes (black arrows) and Kupffer cells (green arrow) with extensive MYD88 cytoplasmic expression. (**Ad**) The PAE + HIR group shows hepatocytes (black arrows) and Kupffer cells (green arrow) with less MYD88 cytoplasmic expression. (**B**) Semi-quantification of the hepatic MYD88 expression. Each value represents the mean ± SEM (*n* = 7). a Significant difference from the sham-operated control group and b significant difference from the HIR group, respectively, at *p* < 0.05.

Further, in the immunohistochemical study for MYD88 (Figure 6A,B), sham and PAE + sham groups displayed hepatocytes and Kupffer cells with negative expression (Figure 6(Aa,Ab)). However, the HIR group showed more cytoplasmic expression in hepatocytes and Kupffer cells compared to the sham-operated control group (Figure 6(Ac)). In addition, less expression in both hepatocytes and Kupffer cells was observed in the HIR group treated with paeonol compared to the HIR untreated group (Figure 6(Ad)).

#### 3.4.2. Effect of Paeonol on Hepatic NF-κB and TNF-α

**Figure 7:**
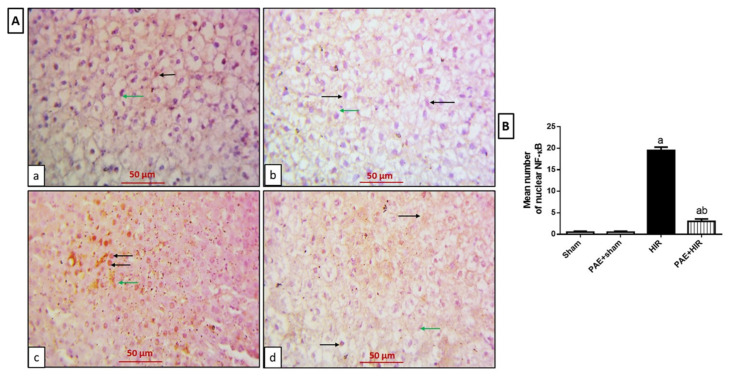
Effect of paeonol (PAE) on nuclear factor-kappa B (NF-κB) in rats’ hepatic ischemia/reperfusion (HIR) injury. (**A**) Photomicrographs of rat liver sections stained for detection of NF-κB (×400). (**Aa**,**Ab**) Sham and PAE + sham groups show negative expression of NF-κB in both hepatocytes (black arrows) and Kupffer cells (green arrows). (**Ac**) The HIR group shows hepatocytes (black arrows) and Kupffer cells (green arrow) with diffused NF-κB nuclear expression. (**Ad**) The PAE + HIR group shows hepatocytes (black arrows) and Kupffer cells (green arrow) with less NF-κB nuclear expression. (**B**) Semi-quantification of the hepatic NF-κB expression. Each value represents the mean ± SEM (*n* = 7). a Significant difference from the sham-operated control group and b significant difference from the HIR group, respectively, at *p* < 0.05.

Furthermore, we investigated the protein expression of the inflammatory mediators NF-κB and TNF-α in hepatic sections. The data depicted in Figure 7A,B for the immunohistochemical study for NF-κB exhibited that in sham and PAE + sham groups, the hepatocytes and Kupffer cells showed a negative expression (Figure 7(Aa,Ab)). However, the HIR group showed diffused NF-κB nuclear expression in hepatocytes and Kupffer cells (Figure 7(Ac)). In contrast, the paeonol-treated HIR group exhibited less NF-κB expression in both hepatocytes and Kupffer cells than noticed in the HIR untreated group (Figure 7(Ad)).

**Figure 8:**
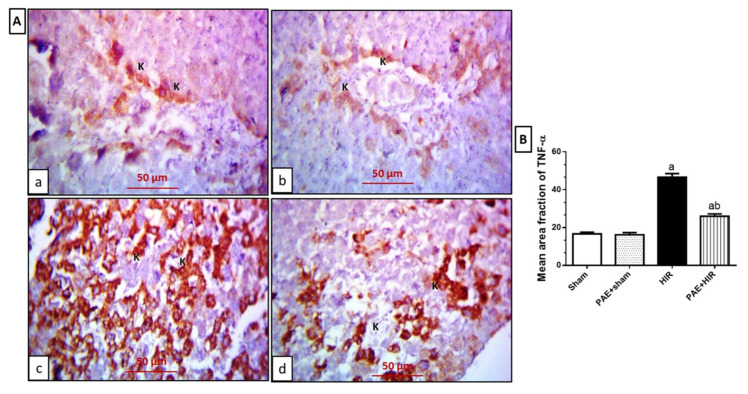
Effect of paeonol (PAE) on tumor necrosis factor-α (TNF-α) in rats’ hepatic ischemia/reperfusion (HIR) injury. (**A**) Photomicrographs of rat liver sections stained for detection of TNF-α (×400). (**Aa**,**Ab**) Sham and PAE + sham groups show Kupffer cells (K) with TNF-α cytoplasmic expression. (**Ac**) The HIR group shows more widespread Kupffer cells (K) with TNF-α cytoplasmic expression in the dilated central vein. (**Ad**) The PAE + HIR group shows fewer Kupffer cells (K) with TNF-α cytoplasmic expression in the central vein. (**B**) Semi-quantification of the hepatic TNF-α expression. Each value represents the mean ± SEM (*n* = 7). a Significant difference from the sham-operated control group and b significant difference from the HIR group, respectively, at *p* < 0.05.

The immunohistochemical study for TNF-α (Figure 8A,B) revealed that sham and PAE + sham groups showed hepatocytes and Kupffer cells with faint TNF-α cytoplasmic expression (Figure 8(Aa,Ab)). On the contrary, the HIR group showed extensive expression in the previously mentioned cells compared to sham and PAE + sham sections (Figure 8(Ac)). Meanwhile, the PAE + HIR group exhibited less expression in both hepatocytes and Kupffer cells compared to the HIR group (Figure 8(Ad)).

### 3.5. Effect of Paeonol on Apoptosis in HIR Injury in Rats

**Figure 9:**
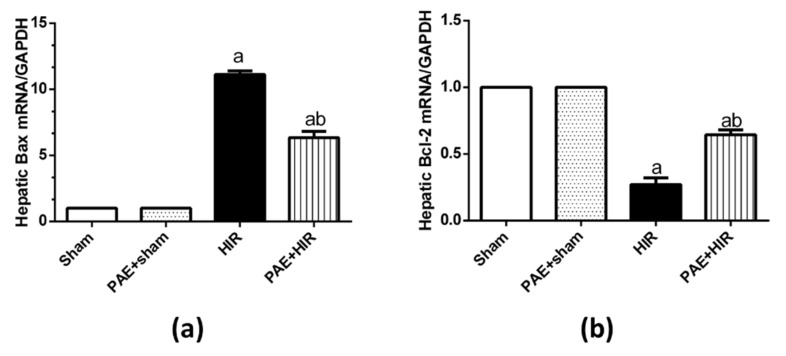
Effect of paeonol (PAE) on apoptosis (Bcl-2-associated X protein (Bax) and B-cell lymphoma 2 (Bcl-2)) in rats’ hepatic ischemia/reperfusion (HIR) injury. The relative expression level of (**a**) Bax and (**b**) Bcl-2 using real-time polymerase chain reaction. Each value represents the mean ± SEM (*n* = 7). a Significant difference from the sham-operated control group and b significant difference from the HIR group, respectively, at *p* ˂ 0.05.

We evaluated the effect of paeonol on the gene expression of the apoptotic molecule Bax and the anti-apoptotic mediator Bcl-2. The data indicated in Figure 9 demonstrates that the relative expression level of Bax was significantly increased in the HIR group compared to the sham-operated control group. However, paeonol pretreatment significantly decreased Bax gene expression compared to HIR untreated rats. Alternatively, HIR significantly decreased the Bcl-2 gene expression compared to the sham-operated control group, and paeonol pretreatment significantly increased this expression. Paeonol did not show any impact on the expression of the indicated genes in the paeonol-treated sham rats.

## 4. Discussion

Paeonol is a natural phenolic compound that has demonstrated potent pharmacological properties against oxidative stress, inflammation, and apoptosis [30,31,32,33,51]. However, the potential role and the underlying mechanisms of paeonol in HIR injury are still undiscovered. The results of the present work demonstrated for the first time that paeonol provided ameliorating effects on HIR injury through suppressing liver oxidative stress, reducing inflammatory reactions, and preventing hepatocellular apoptosis via the activation of the Nrf2/HO-1, and the inhibition of the TLR4/MYD88/NF-κB, and Bax/Bcl-2 signaling pathways. These findings may provide paeonol as a possible protective therapeutic against HIR injury.

For the above goal, we utilized a well-established rat model of HIR injury. Induction of HIR in the current study resulted in an elevation in the serum liver enzymes ALT and AST, pointing to early acute hepatic injury and which are in line with previous reports [52,53], while paeonol pretreatment reduced liver enzyme activities. These data were further confirmed by the histopathological changes. The disturbed lobular architecture and the massive inflammatory cellular infiltration and hemorrhage were dramatically reduced with paeonol administration, indicating that paeonol could mitigate the severity of HIR-induced liver damage. The present results are in agreement with previous studies, which reported that paeonol could ameliorate liver injuries in several animal models [30,51,54] and various experimental I/R injuries [35,36,37].

Oxidative stress, which results from ROS generation along with depletion of antioxidant defense mechanisms, plays a key role in the pathogenesis of HIR injury [5,55,56]. Excess oxygen radicals are produced during the reperfusion of the ischemic hepatocytes, causing lipid peroxidation of cellular membranes, inflammatory cell infiltration, neutrophil stimulation, and hepatic cell damage [57,58]. MDA is a dominant biomarker for lipid peroxidation [59]. On the other hand, NO is well reported to be enhanced in I/R due to the overexpression of NO synthase, resulting in the generation of peroxynitrite, which depletes the antioxidant reserves, induces lipid peroxidation, and causes cellular apoptosis [60]. GSH acts non-enzymatically against oxidative stress by directly scavenging the ROS [61], and SOD is an important member of the antioxidant metalloenzymes that defend against oxidative stress [62]. The results of the current study, in agreement with previous reports [28,63], demonstrated a significant increase in the hepatic MDA and NO levels while a remarkable reduction in the hepatic antioxidant GSH level and SOD activity, proving the contribution of the oxidative stress in the process of HIR injury [28,63]. The pretreatment of paeonol in the rats counterbalanced these alternations, regaining the antioxidant activities and reducing the elevated oxidative biomarkers. Our results are supported by the strong antioxidant efficacy of paeonol which showed hepatoprotective effects in models of lipopolysaccharide/d-galactosamine-induced acute liver failure [51], and diethylnitrosamine-induced hepatocellular carcinoma [64]. These results demonstrate that the prevention of HIR injury by paeonol is partially attributed to its oxidation resistance.

Nrf2 is a crucial endogenous regulator of antioxidant defense mechanism [62]. Under basal conditions, cytoplasmic Keap-1 binds to Nrf2 and restrains its activity through ubiquitination and proteasome-dependent degradation to maintain the cell homeostasis [65]. In response to stress, the redox-sensitive Keap-1 uncouples from Nrf2, allowing the newly synthesized Nrf2 to translocate into the nucleus, where it binds to AREs and forms heterodimers, allowing the recruitment of key transcription factors [66]. HO-1 is an inducible enzyme, and its target gene is regulated by Nrf2. The transcription and expression of HO-1 are mainly considered for the antioxidant defense against oxidative damage [67]. Previous studies have demonstrated the involvement of Nrf2/HO-1 signaling in the regulation of HIR injury [17,68]. In the current data, Nrf2 was significantly enhanced in the HIR group; such enhancement can be explained by the protective, compensatory cellular response of Nrf2 to combat oxidative stress [69]. Our data has pointed out that the elevated Nrf2 levels were probably insufficient to enhance the antioxidant HO-1 protein levels under HIR conditions and are in agreement with previous work [70]. Further, we indicated the ability of paeonol to effectively increase the Nrf2 mRNA, as well as HO-1 protein levels compared to untreated HIR rats, suggesting the contribution of the Nrf2/HO-1 pathway in the protective effects of paeonol on HIR injury, as previously reported for paeonol in other disease models [28,31,71].

Furthermore, HIR injury causes an intricate release of inflammatory mediators and triggers the stimulation of several inflammatory cascades [72]. TNF-α is a well-reported mediator during sterile inflammation in HIR injury that significantly contributes to liver injury [73]. In the present work, paeonol remarkably reduced the number of infiltrating inflammatory cells, as well as TNF-α expression, following previous reports [30]. In addition, it has been previously shown that TNF-α also stimulates neutrophils recruitment via the expression of adhesion molecules and induces chemokines, which further causes the liberation of more ROS and proteases, creating extra injury [74]. Thus, the current data indicates that paeonol displayed an effective anti-inflammatory effect.

The TLR4/NF-κB signaling pathway is a basic pathway that mediates inflammation and plays a crucial role in the ischemic injury of the brain, heart, lung, and liver [22,24,28,75]. Under unstimulated conditions, NF-κB is typically sequestered with IκB in the cytoplasm. In response to a stressful stimulation, IkBα is phosphorylated and deactivated by IκB kinase-β (IKKβ), freeing NF-κB to be translocated to the nucleus and guide the transcription of multiple pro-inflammatory cytokines [76]. TLR4 causes the activation of NF-κB through two downstream pathways, the MYD88-dependent, and independent pathways. The MYD88-dependent pathway leads to the release of multiple inflammatory factors, such as TNF-α, interleukin-1 (IL-1), IL-6, and IL-8, mainly via the stimulation of the NF-κB [77]. In order to detect whether paeonol exerts its anti-inflammatory effects through the TLR4/MYD88/NF-κB signaling pathway, we determined the expression of all of TLR4, MYD88, and NF-κB at the protein level, and the results showed a significant increase in their protein expression in the HIR group compared to the sham-operated rats, while the pretreatment with paeonol dramatically reduced their expression level. The current data were in accordance with the results of Zhai et al., who demonstrated the protective effects of paeonol against the inflammatory response in rheumatoid arthritis via modulating the TLR4/NF-κB pathway in mice [78]. Additionally, Al-Taher et al. revealed an attenuating effect for paeonol in methotrexate-induced cardiac toxicity through inhibiting TLR4/NF-*κ*B/TNF-*α*/IL-6 inflammatory pathway, as well as reducing the pro-apoptotic marker, caspase 3 [45]. Thus, our data proved that paeonol was able to decrease the inflammatory response caused by HIR by inhibiting the TLR4/MYD88/NF-κB signaling pathway.

To determine whether paeonol had an effect on apoptosis stimulated by HIR, we detected the gene expression of Bcl-2 and Bax. Bcl-2 is one of the key anti-apoptotic genes, while Bax is one of the most critical members of apoptotic genes. The level of expression of these Bcl-2 family genes is a reflection of the percentage of apoptosis. Therefore, one possible mechanism by which HIR decreases cell viability is increased ROS level, which then causes an alternation in the expression levels of Bcl-2 family genes. In the current study, the expression level of the anti-apoptotic gene (Bcl-2) was remarkably reduced, and the pro-apoptotic gene (Bax) was significantly elevated in the HIR-induced group, while the administration of paeonol effectively reversed these changes. Another plausible mechanism is the regulation of the two genes by TLR4. Our results are in harmony with previous findings by Li et al. [79]. Moreover, Gong et al. have demonstrated a protective capacity for paeonol against lipopolysaccharide/d-galactosamine-induced acute liver failure in mice via inhibiting Bax, caspases 3, 8, 9, and increasing Bcl-2 [51]. Thus, the current study indicated that paeonol was able to augment mitochondrial activity, suppress ROS production, and inhibit apoptosis by stimulating Bcl-2 and inhibiting Bax gene expressions, thereby displaying a protective role in HIR.

## 5. Conclusions

**Figure 10:**
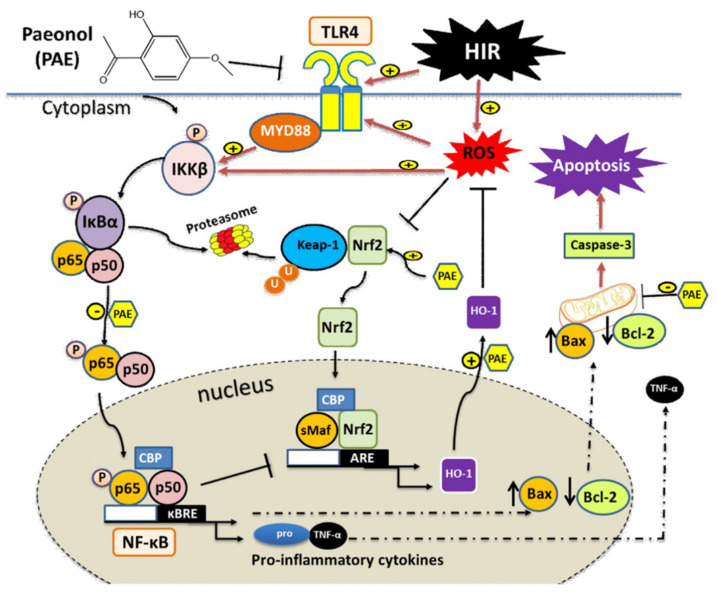
Schematic diagram illustrating the proposed molecular mechanisms underlying the protective effects of paeonol (PAE) in hepatic ischemia/reperfusion (HIR) in rats. (1) Under resting conditions, Kelch-like ECH-associated protein 1 (Keap-1) forms a complex, which leads to the ubiquitination and degradation of nuclear factor erythroid-2-related factor 2 (Nrf2). HIR triggers the increase in reactive oxygen species (ROS), which causes Nrf2 to dissociate from its complex and translocates into the nucleus, where it forms a complex with small Maf proteins (sMaf) and antioxidant response elements (AREs). The formed complex guides the transcription of cytoprotective genes, such as heme-oxygenase-1 (HO-1). PAE augments the Nrf2/HO-1 pathway, while the HIR inhibits its full efficiency. (2) HIR stimulates toll-like receptor 4 (TLR4)/myeloid differentiation primary response gene 88 (MYD88) signaling pathway, which is a critical activator for nuclear factor kappa-B (NF-κB). IκBα, a negative regulator of NF-κB, is phosphorylated and ubiquitinated, allowing for its proteasomal degradation. This leads to freeing the NF-κB subunit and its translocation to the nucleus, where it hetero-dimerizes at the κB response element (κBRE). This further stimulates the transcription of various pro-inflammatory mediators, such as tumor necrosis factor-α (TNF-α) and regulates the pro-apoptotic molecule Bcl-2-associated X protein (Bax) and the anti-apoptotic molecule B-cell lymphoma 2 (Bcl-2). As indicated, PAE inhibits TLR4/MYD88/NF-κB pathways, while HIR enhances their signaling. In addition, PAE inhibits the apoptotic cascade by stimulating Bcl-2, while inhibiting Bax ameliorating HIR-induced apoptosis. Several cross-talks between the three different molecular signaling pathways are illustrated.

The present study demonstrated that paeonol was able to significantly reduce the degree of HIR injury. This hepatoprotective effect was attributable to, at least in part, its antioxidant properties via the modulation of MDA, NO, GSH, and SOD, and the enhancement in Nrf2/HO-1 protein expression, a decrease in TLR4/MYD88/NF-κB inflammatory pathway, and its anti-apoptotic effect via suppressing Bax while activating Bcl-2 gene expressions. The proposed molecular mechanism of action of paeonol and the possible cross-talk between its effectors are illustrated in Figure 10.

## Figures and Tables

**Table 1:** The specific primers for real-time polymerase chain of nuclear factor erythroid-2-related factor 2 (Nrf2), Bcl-2-associated X protein (Bax), B-cell lymphoma 2 (Bcl-2), and glyceraldehyde-3-phosphate dehydrogenase (GAPDH).

Gene	Forward Primer	Reverse Primer
Nrf2	5′-CCCAGCACATCCAGACAGAC-3′	5′-TATCCAGGGCAAGCGACTC-3′
Bax	5′-AGAGGCAGCGGCAGTGAT-3′	5′-AGACACAGTCCAAGGCAGCAG-3′
Bcl-2	5′-TATATGGCCCCAGCATGCGA-3′	5′-GGGCAGGTTTGTCGACCTCA-3′
GAPDH	5′-GTCGGTGTGAACGGATTTG-3′	5′-CTTGCCGTGGGTAGAGTCAT-3′

## Data Availability

The data presented in this study are available in the article.

## References

[1] Weigand K., Brost S., Steinebrunner N., Büchler M., Schemmer P., Müller M. (2012). Ischemia/Reperfusion injury in liver surgery and transplantation: Pathophysiology. HPB Surg..

[2] Guan L.Y., Fu P.Y., Li P.D., Li Z.N., Liu H.Y., Xin M.G., Li W. (2014). Mechanisms of hepatic ischemia-reperfusion injury and protective effects of nitric oxide. World J. Gastrointest. Surg..

[3] Donadon M., Molinari A.F., Corazzi F., Rocchi L., Zito P., Cimino M., Costa G., Raimondi F., Torzilli G. (2016). Pharmacological Modulation of Ischemic-Reperfusion Injury during Pringle Maneuver in Hepatic Surgery. A Prospective Randomized Pilot Study. World J. Surg..

[4] Dar W.A., Sullivan E., Bynon J.S., Eltzschig H., Ju C. (2019). Ischaemia reperfusion injury in liver transplantation: Cellular and molecular mechanisms. Liver Int..

[5] Elias-Miró M., Jiménez-Castro M.B., Rodés J., Peralta C. (2013). Current knowledge on oxidative stress in hepatic ischemia/reperfusion. Free Radic. Res..

[6] Nastos C., Kalimeris K., Papoutsidakis N., Tasoulis M.-K., Lykoudis P.M., Theodoraki K., Nastou D., Smyrniotis V., Arkadopoulos N. (2014). Global consequences of liver ischemia/reperfusion injury. Oxid. Med. Cell. Longev..

[7] Montalvo-Jave E.E., Escalante-Tattersfield T., Ortega-Salgado J.A., Piña E., Geller D.A. (2008). Factors in the pathophysiology of the liver ischemia-reperfusion injury. J. Surg. Res..

[8] Cadenas S. (2018). ROS and redox signaling in myocardial ischemia-reperfusion injury and cardioprotection. Free Radic. Biol. Med..

[9] Abe Y., Hines I.N., Zibari G.B., Pavlick K., Gray L., Kitagawa Y., Grisham M.B. (2009). Mouse model of liver ischemia and reperfusion injury: Method for studying reactive oxygen and nitrogen metabolites in vivo. Free Radic. Biol. Med..

[10] Zhang S., Feng Z., Gao W., Duan Y., Fan G., Geng X., Wu B., Li K., Liu K., Peng C. (2020). Aucubin Attenuates Liver Ischemia-Reperfusion Injury by Inhibiting the HMGB1/TLR-4/NF-κB Signaling Pathway, Oxidative Stress, and Apoptosis. Front. Pharmacol..

[11] Ibrahim M.A., Abdel-Gaber S.A., Amin E.F., Ibrahim S.A., Mohammed R.K., Abdelrahman A.M. (2014). Molecular mechanisms contributing to the protective effect of levosimendan in liver ischemia-reperfusion injury. Eur. J. Pharmacol..

[12] Morsy M.A. (2011). Protective effect of lisinopril on hepatic ischemia/reperfusion injury in rats. Indian J. Pharmacol..

[13] Ikeda T., Yanaga K., Kishikawa K., Kakizoe S., Shimada M., Sugimachi K. (1992). Ischemic injury in liver transplantation: Difference in injury sites between warm and cold ischemia in rats. Hepatology.

[14] Scheenstra M.R., De Cuyper I.M., Branco-Madeira F., de Bleser P., Kool M., Meinders M., Hoogenboezem M., Mul E., Wolkers M.C., Salerno F. (2016). GATA1-Deficient Dendritic Cells Display Impaired CCL21-Dependent Migration toward Lymph Nodes Due to Reduced Levels of Polysialic Acid. J. Immunol..

[15] Liu S., Khemlani L.S., A Shapiro R., Johnson M.L., Liu K., A Geller D., Watkins S.C., Goyert S.M., Billiar T.R. (1998). Expression of CD14 by hepatocytes: Upregulation by cytokines during endotoxemia. Infect. Immun..

[16] Ding Y., Liu P., Chen Z.-L., Zhang S.-J., Wang Y.-Q., Cai X., Luo L., Zhou X., Zhao L. (2018). Emodin Attenuates Lipopolysaccharide-Induced Acute Liver Injury via Inhibiting the TLR4 Signaling Pathway in vitro and in vivo. Front. Pharmacol..

[17] Yu Q., Chen S., Tang H., Zhang X., Tao R., Yan Z., Shi J., Guo W., Zhang S. (2021). Veratric acid alleviates liver ischemia/reperfusion injury by activating the Nrf2 signaling pathway. Int. Immunopharmacol..

[18] Bardallo R.G., Panisello-Roselló A., Sanchez-Nuno S., Alva N., Roselló-Catafau J., Carbonell T. (2021). Nrf2 and oxidative stress in liver ischemia/reperfusion injury. FEBS J..

[19] Hua Z., Hou B. (2013). TLR signaling in B-cell development and activation. Cell. Mol. Immunol..

[20] Li P.M., Li Y.L., Liu B., Wang W.J., Wang Y.Z., Li Z. (2014). Curcumin inhibits MHCC97H liver cancer cells by activating ROS/TLR-4/caspase signaling pathway. Asian Pac. J. Cancer Prev..

[21] Li Y., Deng S.-L., Lian Z.-X., Yu K. (2019). Roles of Toll-Like Receptors in Nitroxidative Stress in Mammals. Cells.

[22] Chen H., Zhang R.Q., Wei X.G., Ren X.M., Gao X.Q. (2016). Mechanism of TLR-4/NF-κB pathway in myocardial ischemia reperfusion injury of mouse. Asian Pac. J. Trop. Med..

[23] Kadono K., Uchida Y., Hirao H., Miyauchi T., Watanabe T., Iida T., Ueda S., Kanazawa A., Mori A., Okajima H. (2017). Thrombomodulin Attenuates Inflammatory Damage Due to Liver Ischemia and Reperfusion Injury in Mice in Toll-Like Receptor 4-Dependent Manner. Am. J. Transplant..

[24] Liu X., Zhang X., Wang F., Liang X., Zeng Z., Zhao J., Zheng H., Jiang X., Zhang Y. (2017). Improvement in cerebral ischemia-reperfusion injury through the TLR4/NF-κB pathway after Kudiezi injection in rats. Life Sci..

[25] Qi M., Zheng L., Qi Y., Han X., Xu Y., Xu L., Yin L., Wang C., Zhao Y., Sun H. (2015). Dioscin attenuates renal ischemia/reperfusion injury by inhibiting the TLR4/MyD88 signaling pathway via up-regulation of HSP70. Pharmacol. Res..

[26] Tao X., Sun X., Xu L., Yin L., Han X., Qi Y., Xu Y., Zhao Y., Wang C., Peng J. (2016). Total Flavonoids from Rosa laevigata Michx Fruit Ameliorates Hepatic Ischemia/Reperfusion Injury through Inhibition of Oxidative Stress and Inflammation in Rats. Nutrients.

[27] Gao Y., Li Z.T., Jin L., Lin J., Fan Z.L., Zeng Z., Huang H.F. (2021). Melatonin attenuates hepatic ischemia-reperfusion injury in rats by inhibiting NF-κB signaling pathway. Hepatobiliary Pancreat. Dis. Int..

[28] Morsy M.A., Abdel-Gaber S.A., Rifaai R.A., Mohammed M.M., Nair A.B., Abdelzaher W.Y. (2022). Protective mechanisms of telmisartan against hepatic ischemia/reperfusion injury in rats may involve PPARγ-induced TLR4/NF-κB suppression. Biomed. Pharmacother..

[29] Du P., Zhang X., Luo K., Li Y., Fu C., Xiao J., Xiao Q. (2022). Curculigoside mitigates hepatic ischemia/reperfusion-induced oxidative stress, inflammation, and apoptosis via activation of the Nrf-2/HO-1 pathway. Hum. Exp. Toxicol..

[30] Ding Y., Li Q., Xu Y., Chen Y., Deng Y., Zhi F., Qian K. (2016). Attenuating Oxidative Stress by Paeonol Protected against Acetaminophen-Induced Hepatotoxicity in Mice. PLoS ONE.

[31] Li H., Song F., Duan L.R., Sheng J.J., Xie Y.H., Yang Q., Chen Y., Dong Q.Q., Zhang B.L., Wang S.W. (2016). Paeonol and danshensu combination attenuates apoptosis in myocardial infarcted rats by inhibiting oxidative stress: Roles of Nrf2/HO-1 and PI3K/Akt pathway. Sci. Rep..

[32] Jin X., Wang J., Xia Z.-M., Shang C.-H., Chao Q.-L., Liu Y.-R., Fan H.-Y., Chen D.-Q., Qiu F., Zhao F. (2016). Anti-inflammatory and Anti-oxidative Activities of Paeonol and Its Metabolites Through Blocking MAPK/ERK/p38 Signaling Pathway. Inflammation.

[33] Hafez H., Morsy M., Mohamed M., Zenhom N. (2019). Mechanisms underlying gastroprotective effect of paeonol against indomethacin-induced ulcer in rats. Hum. Exp. Toxicol..

[34] Al-Taher A.Y., Morsy M.A., Rifaai R.A., Zenhom N.M., Abdel-Gaber S.A. (2020). Paeonol Attenuates Methotrexate-Induced Cardiac Toxicity in Rats by Inhibiting Oxidative Stress and Suppressing TLR4-Induced NF-κB Inflammatory Pathway. Mediators Inflamm..

[35] Liao W.-Y., Tsai T.-H., Ho T.-Y., Lin Y.-W., Cheng C.-Y., Hsieh C.-L. (2016). Neuroprotective Effect of Paeonol Mediates Anti-Inflammation via Suppressing Toll-Like Receptor 2 and Toll-Like Receptor 4 Signaling Pathways in Cerebral Ischemia-Reperfusion Injured Rats. Evid.-Based Complement. Altern. Med..

[36] Ma L., Chuang C.-C., Weng W., Zhao L., Zheng Y., Zhang J., Zuo L. (2016). Paeonol Protects Rat Heart by Improving Regional Blood Perfusion during No-Reflow. Front. Physiol..

[37] Mohamed M.Z., Morsy M.A., Mohamed H.H., Hafez H.M. (2020). Paeonol protects against testicular ischaemia-reperfusion injury in rats through inhibition of oxidative stress and inflammation. Andrologia.

[38] Buege J.A., Aust S.D. (1978). Microsomal lipid peroxidation. Methods Enzymol..

[39] Sastry K.V., Moudgal R.P., Mohan J., Tyagi J.S., Rao G.S. (2002). Spectrophotometric determination of serum nitrite and nitrate by copper-cadmium alloy. Anal. Biochem..

[40] Moron M.S., Depierre J.W., Mannervik B. (1979). Levels of glutathione, glutathione reductase and glutathione S-transferase activities in rat lung and liver. Biochim. Biophys. Acta.

[41] Marklund S., Marklund G. (1974). Involvement of the superoxide anion radical in the autoxidation of pyrogallol and a convenient assay for superoxide dismutase. Eur. J. Biochem..

[42] Abdel-Gaber S.A., Geddawy A., Moussa R.A. (2019). The hepatoprotective effect of sitagliptin against hepatic ischemia reperfusion-induced injury in rats involves Nrf-2/HO-1 pathway. Pharmacol. Rep..

[43] Horiuchi M., Itoh A., Pleasure D., Itoh T. (2006). MEK-ERK Signaling Is Involved in Interferon-γ-induced Death of Oligodendroglial Progenitor Cells. J. Biol. Chem..

[44] Refaie M.M.M., El-Hussieny M., Zenhom N.M. (2018). Protective role of nebivolol in cadmium-induced hepatotoxicity via downregulation of oxidative stress, apoptosis and inflammatory pathways. Environ. Toxicol. Pharmacol..

[45] Wimmer I., Tröscher A.R., Brunner F., Rubino S.J., Bien C.G., Weiner H.L., Lassmann H., Bauer J. (2018). Systematic evaluation of RNA quality, microarray data reliability and pathway analysis in fresh, fresh frozen and formalin-fixed paraffin-embedded tissue samples. Sci. Rep..

[46] VanGuilder H.D., Vrana K.E., Freeman W.M. (2008). Twenty-five years of quantitative PCR for gene expression analysis. Biotechniques.

[47] Bancroft J.D., Gamble M. (2007). Theory and Practice of Histological Techniques.

[48] Ahmed A.-S.F., Bayoumi A.M., Eltahir H.M., Hafez S.M.A., Abouzied M.M. (2020). Amelioration of Sepsis-induced liver and lung injury by a superoxide dismutase mimetic; role of TNF-α and Caspase-3. J. Adv. Biomed. Pharm. Sci..

[49] Samuhasaneeto S., Thong-Ngam D., Kulaputana O., Suyasunanont D., Klaikeaw N. (2009). Curcumin decreased oxidative stress, inhibited NF-κB activation, and improved liver pathology in ethanol-induced liver injury in rats. J. Biomed. Biotechnol..

[50] Du Y., Qian B., Gao L., Tan P., Chen H., Wang A., Zheng T., Pu S., Xia X., Fu W. (2019). Aloin Preconditioning Attenuates Hepatic Ischemia/Reperfusion Injury via Inhibiting TLR4/MyD88/NF-κB Signal Pathway In Vivo and In Vitro. Oxid. Med. Cell. Longev..

[51] Gong X., Yang Y., Huang L., Zhang Q., Wan R.Z., Zhang P., Zhang B. (2017). Antioxidation, anti-inflammation and anti-apoptosis by paeonol in LPS/d-GalN-induced acute liver failure in mice. Int. Immunopharmacol..

[52] Ko J.S., Gwak M.S., Kim G.S., Shin Y.H., Ryu S., Kim J.S., Kim S.J., Kim S.T. (2013). The protective effect of ischemic preconditioning against hepatic ischemic-reperfusion injury under isoflurane anesthesia in rats. Transplant. Proc..

[53] Kim D., Choi J.W., Han S., Gwak M.S., Kim G.S., Jeon S.Y., Ryu S., Hahm T.S., Ko J.S. (2020). Ischemic Preconditioning Protects Against Hepatic Ischemia-Reperfusion Injury Under Propofol Anesthesia in Rats. Transplant. Proc..

[54] Sun X., Wang P., Yao L.P., Wang W., Gao Y.M., Zhang J., Fu Y.J. (2018). Paeonol alleviated acute alcohol-induced liver injury via SIRT1/Nrf2/NF-κB signaling pathway. Environ. Toxicol. Pharmacol..

[55] Jiang Y., He X., Simonaro C.M., Yi B., Schuchman E.H. (2021). Acid Ceramidase Protects Against Hepatic Ischemia/Reperfusion Injury by Modulating Sphingolipid Metabolism and Reducing Inflammation and Oxidative Stress. Front. Cell Dev. Biol..

[56] Li Z., Wang Y., Zhang Y., Wang X., Gao B., Li Y., Li R., Wang J. (2021). Protective Effects of Fisetin on Hepatic Ischemia-reperfusion Injury Through Alleviation of Apoptosis and Oxidative Stress. Arch. Med. Res..

[57] Peralta C., Jiménez-Castro M.B., Gracia-Sancho J. (2013). Hepatic ischemia and reperfusion injury: Effects on the liver sinusoidal milieu. J. Hepatol..

[58] Konishi T., Lentsch A.B. (2017). Hepatic Ischemia/Reperfusion: Mechanisms of Tissue Injury, Repair, and Regeneration. Gene Expr..

[59] Ayala A., Muñoz M.F., Argüelles S. (2014). Lipid peroxidation: Production, metabolism, and signaling mechanisms of malondialdehyde and 4-hydroxy-2-nonenal. Oxid. Med. Cell. Longev..

[60] Pacher P., Beckman J.S., Liaudet L. (2007). Nitric oxide and peroxynitrite in health and disease. Physiol. Rev..

[61] Franco R., Schoneveld O.J., Pappa A., Panayiotidis M.I. (2007). The central role of glutathione in the pathophysiology of human diseases. Arch. Physiol. Biochem..

[62] Xu G., Wang X., Xiong Y., Ma X., Qu L. (2019). Effect of sevoflurane pretreatment in relieving liver ischemia/reperfusion-induced pulmonary and hepatic injury. Acta Cir. Bras..

[63] Kou X., Zhu J., Xie X., Hao M., Zhao Y. (2020). The protective effect of glycyrrhizin on hepatic ischemia-reperfusion injury in rats and possible related signal pathway. Iran. J. Basic Med. Sci..

[64] Chen B., Ning M., Yang G. (2012). Effect of paeonol on antioxidant and immune regulatory activity in hepatocellular carcinoma rats. Molecules.

[65] Song M.Y., Lee D.Y., Chun K.S. (2021). The Role of NRF2/KEAP1 Signaling Pathway in Cancer Metabolism. Int. J. Mol. Sci..

[66] Baird L., Yamamoto M. (2020). The Molecular Mechanisms Regulating the KEAP1-NRF2 Pathway. Mol. Cell. Biol..

[67] Ryter S.W. (2019). Heme oxygenase-1/carbon monoxide as modulators of autophagy and inflammation. Arch. Biochem. Biophys..

[68] Zhao Y., Kong G.Y., Pei W.M., Zhou B., Zhang Q.Q., Pan B.B. (2019). Dexmedetomidine alleviates hepatic injury via the inhibition of oxidative stress and activation of the Nrf2/HO-1 signaling pathway. Eur. Cytokine Netw..

[69] Li C.Q., Kim M.Y., Godoy L.C., Thiantanawat A., Trudel L.J., Wogan G.N. (2009). Nitric oxide activation of Keap1/Nrf2 signaling in human colon carcinoma cells. Proc. Natl. Acad. Sci. USA.

[70] Zhong Q., Mishra M., Kowluru R.A. (2013). Transcription factor Nrf2-mediated antioxidant defense system in the development of diabetic retinopathy. Investig. Ophthalmol. Vis. Sci..

[71] Wu J., Xu L., Sun C., Zhang B., Li J., Sun J., Zhang Y., Sun D. (2017). Paeonol alleviates epirubicin-induced renal injury in mice by regulating Nrf2 and NF-κB pathways. Eur. J. Pharmacol..

[72] Jaeschke H., Farhood A., Bautista A.P., Spolarics Z., Spitzer J.J. (1993). Complement activates Kupffer cells and neutrophils during reperfusion after hepatic ischemia. Am. J. Physiol..

[73] Perry B.C., Soltys D., Toledo A.H., Toledo-Pereyra L.H. (2011). Tumor necrosis factor-α in liver ischemia/reperfusion injury. J. Investig. Surg..

[74] Casillas-Ramírez A., Mosbah I.B., Ramalho F., Roselló-Catafau J., Peralta C. (2006). Past and future approaches to ischemia-reperfusion lesion associated with liver transplantation. Life Sci..

[75] Sharma A.K., LaPar D.J., Stone M.L., Zhao Y., Kron I.L., Laubach V.E. (2013). Receptor for advanced glycation end products (RAGE) on iNKT cells mediates lung ischemia-reperfusion injury. Am. J. Transplant..

[76] Somade O.T., Ajayi B.O., Safiriyu O.A., Oyabunmi O.S., Akamo A.J. (2019). Renal and testicular up-regulation of pro-inflammatory chemokines (RANTES and CCL2) and cytokines (TNF-α, IL-1β, IL-6) following acute edible camphor administration is through activation of NF-kB in rats. Toxicol. Rep..

[77] Zhao H., Perez J.S., Lu K., George A., Ma D. (2014). Role of Toll-like receptor-4 in renal graft ischemia-reperfusion injury. Am. J. Physiol. Physiol..

[78] Zhai K.F., Duan H., Luo L., Cao W.G., Han F.K., Shan L.L., Fang X.M. (2017). Protective effects of paeonol on inflammatory response in IL-1β-induced human fibroblast-like synoviocytes and rheumatoid arthritis progression via modulating NF-κB pathway. Inflammopharmacology.

[79] Li X., Wang L., Yang X., Huang C. (2020). Metformin attenuates ischemia-reperfusion injury of fatty liver in rats through inhibition of the TLR4/NF-κB axis. Balk. Med. J..

